# NAVIGATING HEALTH AND IDENTITY TO PROMOTE FORTITUDE AMONG SGM PEOPLE: INSIGHTS FROM AGING WITH PRIDE

**DOI:** 10.1093/geroni/igae098.2191

**Published:** 2024-12-31

**Authors:** Charles Hoy-Ellis, Karen Fredriksen-Goldsen

**Affiliations:** Chair; Discussant

## Abstract

This symposium showcases groundbreaking research illustrating the multifaceted nature of fortitude among sexual and gender minority (SGM) older adults, focusing on health, digital health access, generativity, and intersectionality. The collection of studies in this symposium leverages cross-sectional and longitudinal data from Aging with Pride: National, Health, Aging and Sexuality/Gender Study (NHAS) to address critical gaps in our understanding of the unique strengths in this population despite the adversity they face. The first presentation illuminates the disproportionate experiences of violence and abuse among older transgender adults, revealing significant links between intimate partner violence (IPV), victimization, internalized stigma, and adverse mental health outcomes. It spotlights the need for interventions that target these risk factors and promote fortitude. The second presentation examines the digital divide, exploring how older SGM adults utilize health information technologies. By comparing usage patterns and identifying barriers and facilitators, this research emphasizes the importance of improving health care access. The third presentation will examine the predictors of generativity among SGM older adults, highlighting how psychological and social resources can mitigate stigma. This study offers valuable perspectives on fostering intergenerational support and community engagement within SGM communities. Lastly, the symposium will feature research examining the intersectionality of identity among SGM older adults, showcasing heterogeneity among this population by identifying patterns based on identity centrality and discrimination experiences. Highlighting the impacts of recent policy shifts targeting SGM communities, these studies collectively underscore the crucial need for resilience-enhancing strategies that incorporate intersectional approaches for promoting health equity among vulnerable, at-risk populations.

